# Gut Microbiota-Based Pharmacokinetics and the Antidepressant Mechanism of Paeoniflorin

**DOI:** 10.3389/fphar.2019.00268

**Published:** 2019-03-20

**Authors:** Jin-Bo Yu, Zhen-Xiong Zhao, Ran Peng, Li-Bin Pan, Jie Fu, Shu-Rong Ma, Pei Han, Lin Cong, Zheng-Wei Zhang, Li-Xin Sun, Jian-Dong Jiang, Yan Wang

**Affiliations:** ^1^Department of Traditional Chinese Medicine, School of Chinese Materia Medica, Shenyang Pharmaceutical University, Shenyang, China; ^2^State Key Laboratory of Bioactive Substance and Function of Natural Medicines Institute of Materia Medica, Chinese Academy of Medical Sciences and Peking Union Medical College, Beijing, China; ^3^Department of Pharmaceutical Analysis, School of Pharmacy, Shenyang Pharmaceutical University, Shenyang, China

**Keywords:** paeoniflorin, pharmacokinetics, depression, gut microbiota, carboxylesterase, benzoic acid

## Abstract

Paeoniflorin, the main component of Xiaoyao Wan, presents low oral bioavailability and unclear antidepressant mechanism. To elucidate the potential reasons for the low bioavailability of paeoniflorin and explore its antidepressant mechanism from the perspective of the gut microbiota, here, a chronic unpredictable depression model and forced swimming test were firstly performed to examine the antidepressant effects of paeoniflorin. Then the pharmacokinetic study of paeoniflorin in rats was performed based on the gut microbiota; meanwhile, the gut microbiota incubated with paeoniflorin *in vitro* was used to identify the possible metabolites of paeoniflorin. Molecular virtual docking experiments together with the specific inhibitor tests were applied to investigate the mechanism of paeoniflorin metabolism by the gut microbiota. Finally, the intestinal microbiota composition was analyzed by 16S rRNA gene sequencing technology. The pharmacodynamics tests showed that paeoniflorin had significant antidepressant activity, but its oral bioavailability was 2.32%. Interestingly, we found paeoniflorin was converted into benzoic acid by the gut microbiota, and was mainly excreted through the urine with the gut metabolite benzoic acid as the prominent excreted form. Moreover, paeoniflorin could also regulate the composition of the gut microbiota by increasing the abundance of probiotics. Therefore, the metabolism effect of gut microbiota may be one of the main reasons for the low oral bioavailability of paeoniflorin. Additionally, paeoniflorin can be metabolized into benzoic acid via gut microbiota enzymes, which might exert antidepressant effects through the blood–brain barrier into the brain.

## Introduction

Depression is a common disease that seriously impairs social function. Depression, which is preceded only by cardiovascular disease, is expected to become the second largest threat to human health by 2020 (Nestler et al., 2002; Dennis and Allen, 2008). At present, the underlying mechanism of depression remains unclear. In addition to the classical monoamine neurotransmitter theory (Blier, 2003), recent studies have confirmed that the hypothesis of synaptic plasticity (Schatzberg, 2015; Nanou and Catterall, 2018), epigenetics (Saavedra et al., 2016), the immune system (Jiang et al., 2017), and astrocytes (Wang et al., 2018) play an essential role in the pathogenesis of depression (Zhuo et al., 2017). Although many hypotheses have been proposed regarding the mechanism, the pathogenesis of depression has not been fully explained, making accurate, effective diagnosis, and treatment more difficult.

The gut microbiota, which accounts for a large proportion of the human body, is considered the largest and most direct external human body environment. A growing number of studies have shown that the gut microbiota is widely involved in metabolic syndrome, autoimmune diseases, emotional cognitive dysfunction and other diseases, and have important effects on and significance for human health and diseases. The gut microbiota also plays an important role in human central nervous system diseases (Wang and Kasper, 2014), affecting the normal development of the nervous system as well as participated in the pathogenesis of many mental illnesses; furthermore, the gut microbiota could regulate anxiety, mood, cognition, and pain (Mohajeri et al., 2018).

Xiaoyao Wan, a relatively common Chinese patent medicine, whose precursor is Xiaoyao San from the Preions of the Bureau of Taiping People’s Welfare Pharmacy of the Song Dynasty, mainly comprises eight herbs such as *Bupleuri Radix, Angelica sinensis Radix, Paeoniae Radix Alba, Atractylodis Macrocephalae Rhizome, Poria, Glycyrrhizae Radix Et Rhizoma, Menthae Haplocalycis Herba*, and *Zingiberis Rhizoma Recens*. Xiaoyao Wan soothes the liver, strengths the spleen, nourishes the blood and regulates menstruation. In addition, Xiaoyao Wan is mainly used to treat depression, chest pain, dizziness, loss of appetite, irregular menstruation caused by liver stagnation and spleen deficiency in clinical settings (Chinese Pharmacopoeia Commission, 2015). Moreover, Xiaoyao Wan has been widely used in the treatment of various forms of depressions over the past 100 years. Paeoniflorin, the main component of Xiaoyao Wan according to our previous studies about Xiaoyao Wan, is a potential drug candidate for the treatment of Parkinson’s and Alzheimer’s disease (Lan et al., 2013; Wang et al., 2014; Gu et al., 2016; Zheng et al., 2017), and has antidepressant activity. Based on recent studies, the possible mechanisms of paeoniflorin in the treatment of depression include increasing monoamine neurotransmitter levels, modulating HPA axis dysfunction, repairing damaged neurons, inhibiting monoamine oxidase expression, and enhancing neuroprotection (Mao et al., 2009; Guo et al., 2012; Qiu F. et al., 2013; Qiu F.M. et al., 2013). However, the poor oral absorption and the low bioavailability of paeoniflorin could be considerably difficult to explain the underlying mechanism of the antidepressant effects of paeoniflorin.

In fact, low bioavailability is a common problem for many effective natural compounds. As a part of our continuous investigation of natural products based on gut microbiota (Feng et al., 2015, 2018; Wang et al., 2015, 2017a,b; Zhao et al., 2018), the present study displayed that degradation by gut microbiota enzymes may be one of the main reasons for the low bioavailability of paeoniflorin. Benzoic acid, a characteristic metabolite of paeoniflorin from the gut bacteria, can penetrate the BBB and may exert antidepressant effects in brain. Here, we provide a certain reference value for the antidepressant mechanism of natural products (such as paeoniflorin) possessing low bioavailability which might be associated with the gut microbiota-based pharmacokinetics.

## Materials and Methods

### Chemicals and Reagents

Paeoniflorin was purchased from Solaibao Biotechnology, Co., Ltd. (Beijing, China). Chloramphenicol and benzoic acid were provided by the National Institutes for Food and Drug Control (Beijing, China). BNPP and fluoxetine were also supplied by Solabao Biotechnology, Co., Ltd. The purity of the above chemicals is greater than 98% (HPLC). Chromatography grade methanol and acetonitrile were obtained from Thermo Fisher Scientific, Co., Ltd. (Fair Lawn, NJ, United States). Other chromatographic reagents were obtained from domestic reagent companies. In addition, *Bifidobacterium longum* (*B. longum*), *Lactobacillus acidophilus* (*L. acidophilus*), *Clostridium butyricum* (*C. butyricum*), *Bifidobacterium breve* (*B. breve*), *Lactobacillus casei* (*L. casei*), *Staphylococcus aureus* (*S. aureus*), *Staphylococcus epidermidis* (*S. epidermidis), Enterobacter aerogenes* (*E. aerogenes*), *Escherichia coli* (*E. coli*), *Proteus vulgaris* (*P. vulgaris*), *Enterobacter cloacae* (*E. cloacae*), *Enterococcus cecorum* (*E. cecorum*), *Proteus mirabilis* (*P. mirabilis*), *Klebsiella pneumoniae* (*K. pneumonia), Shigella boydii* (*Sh. Boydii*), *Enterococcus faecium* (*E. faecium*), *Pseudomonas aeruginosa* (*P. aeruginosa*), and *Enterococcus faecalis* (*E. faecalis*) were purchased from Nanjing Bianzhen Biotechnology, Co., Ltd. (Nanjing, China). TIANamp Bacteria DNA Kit was obtained from TIANGEN BIOTECH, Co., Ltd. (Beijing, China). DL 5000 DNA marker was provided by Takara Biomedical Technology, Co., Ltd. (Beijing, China).

### Instruments and Methods

Liquid chromatography with tandem mass spectrometry (LC-MS/MS 8050, Shimadzu Corporation, Kyoto, Japan) was applied to quantitatively determine paeoniflorin and benzoic acid. Chromatography separation was performed using an XSS T3 column (3.5 μm, 2.1 mm × 100 mm, Waters, Milford, MA, United States) with the column temperature maintained at 40°C at a flow rate of 0.4 mL/min. The mobile phase was water containing 0.05% acetic acid (eluent A) with methanol (eluent B). The gradient was as follows: 25% eluent B for 2 min; 25–85% B from 2 to 4 min; 85–25% B from 4 to 5 min and held at 25% from 5 to 7 min. The autosampler temperature was set to 4°C. Mass spectrometry was carried out using multiple reaction monitoring mode (MRM). The following product ion precursors were monitored: paeoniflorin (m/z) was 502.95 [M+Na]^+^→341.05, benzoic acid (m/z) was 121.10 [M-H]^-^→77.10 and chloramphenicol (m/z) was 321.15 [M-H]^-^→152.00 which was performed as internal standard (IS). The nebulizing gas flow rate was 2.8 L/min, the drying gas flow rate was 10 L/min, and the heating gas flow rate was 10 L/min.

Ultra-performance liquid chromatography (UFLC XR, Shimadzu Corporation, Japan) was performed to quantitatively determine paeoniflorin in Xiaoyao Wan. Chromatography was performed using an Alltima C_18_ column (5 μm, 4.6 mm × 250 mm, Alltech, Deerfield, IL, United States) maintained at 40°C. Water containing 0.05% acetic acid and methanol were used as mobile phases A and B with a flow rate of 0.8 mL/min. The gradient elution procedure was 0.01–15 min, 30% (B); 15–20 min, 30–45% (B); and 20–25 min, 45-65% (B). The detection wavelength was set at 231 nm.

### Animals

Male Sprague-Dawley (SD) rats (180–200 g) were supplied by Institute of Laboratory Animal Science, Chinese Academy of Medical Sciences (Beijing, China). The animals were placed in a room with a temperature of 22–24°C, a humidity of 45%, and a 12-h light/dark cycle (light time from 8:00 to 20:00). Before the experiment, rats were fasted for 12 h but had free access to water. All experiments were conducted in accordance with institutional and ethics guidelines and were approved by the Laboratories Institutional Animal Care and Use Committee of the Chinese Academy of Medical Sciences and Peking Union Medical College.

### Determination of Paeoniflorin in Xiaoyao Wan

Xiaoyao Wan was extracted according to the methods of the other studies (Liang and Shi, 2011). UFLC method was applied to detect the content of paeoniflorin in Xiaoyao Wan.

### Antidepressant Activity of Paeoniflorin

#### Chronic Unpredictable Stress Depression Model

Forty rats were randomly divided into the following groups (*n* = 8): the normal control group, the model control group, the fluoxetine group (10 mg/kg), the paeoniflorin low-dose group (10 mg/kg), and the paeoniflorin high-dose group (20 mg/kg). Except for the blank control group, the groups were randomly exposed to two stressors, including behavioral constraints, ice water swimming, electric shock, cold stimulation, clip tail, wet bedding, light/dark reversed, shaking, foods and water deprivation, continuously for 8 weeks to establish a chronic unpredictable stress model. The sucrose preference test was performed at 0 and 14 days after administration.

#### Forced Swimming Test

Fifteen rats were randomly assigned to the following groups (*n* = 5): the normal control group, the paeoniflorin low-dose group (10 mg/kg), the paeoniflorin high-dose group (20 mg/kg). A forced swimming test was carried out according to published research and we made appropriate adjustments (Tang et al., 2015). Each rat was placed individually in a transparent circular cylinder with a water depth of approximately 25 cm and the temperature was kept at 25 ± 3°C. The water used was changed before performing each forced swimming test to avoid influencing the experimental rats. Each rat was pretested for 15 min before administration, then, rats were removed and placed in a cage to dry for continuous searing. After 24 h, rats were administered paeoniflorin; after 1 h, they were exposed to the same experimental conditions outlined above for a 6-min process. Immobility behaviors in the last 4 min were recorded when the rats showed only the necessary movements to keep their heads above the water without swimming and/or climbing action.

### Pharmacokinetics of Paeoniflorin in SD Rats

Six SD rats were orally administered of paeoniflorin (20 mg/kg). Blood (200 μL) from the femoral vein was collected into a heparinized tube at 0.00, 0.08, 0.17, 0.33, 0.5, 0.75, 1, 2, 4, 6, 10, and 12 h after drug administration.

Five SD rats were intravenously administered of paeoniflorin (2 mg/kg). Blood (200 μL) from the femoral vein was collected into a heparinized tube at 0.00, 0.03, 0.08, 0.17, 0.25, 0.33, 0.5, 0.75, 1, 2, 4, and 6 h after administration.

Plasma samples were immediately stored at 4°C until analysis. The method validation assays were carried out in terms of specificity, linearity, sensitivity, accuracy, precisions, recovery, matrix effect and stability.

### Biotransformation of Paeoniflorin *in vitro*

#### Metabolism of Paeoniflorin by Intestinal Bacteria *in vitro*

The colon contents of six rats were pooled, and 5 g of the sample was transferred to a ziplock bag and then mixed thoroughly with anaerobic medium (100 mL). After mixing completely, the cultures were stored at 37°C and preincubated for 1 h. Then, 10 μL of paeoniflorin (100 mg/mL) was added to fresh rat intestinal microbial culture (990 μL) under anaerobic conditions of N_2_, with a final concentration of 1 mg/mL of paeoniflorin in the culture system (Hattori et al., 1988). The inactivated bacteria solution was used as a negative control. Cultures were incubated at 37°C for 0, 2, 6, 12, and 24 h, respectively. After terminated the reaction with 3 volumes of acetonitrile, samples were vortexed for 30 s and then centrifuged at 16,162 *g* for 5 min. A total of 100 μL of the supernatant were transferred to a centrifuge tube, centrifuged at 16,162 *g* for 5 min and filtered through a 0.45 μm microporous membrane. Finally, 5 μL of the filtrate solution was collected for analysis.

#### Metabolism of Paeoniflorin in Liver Microsome and the Homogenate of the Liver Tissue *in vitro*

Samples were incubated at 37°C in a shaker. The incubation mixtures were 0.2 mL of the final volume and the protein concentration was 1 mg/mL. Paeoniflorin was dissolved in methanol before it was added to the incubation mixtures in the final concentration of 5 μM and the final methanol concentration did not exceed 1%. The reaction was initiated by the addition of NADPH after a 3 min preincubation at 37°C.

Fresh rat liver tissue was cut into small pieces, 0.05 M Tris-HC1 buffer (1:2, pH 7.4) was added. The mixture was homogenized in a mixer and paeoniflorin was added to the liver homogenate at a final concentration of 100 μg/mL. Then, the mixture was incubated at 37°C for 1 h.

The above reactions were terminated by the addition of 0.4 mL acetonitrile (1:2). Proteins were precipitated by centrifugation at 10,625 *g* for 5 min, and 10 μL supernatant was injected into the UPLC system for quantification.

### Pharmacokinetics of Paeoniflorin Mediated by the Gut Microbiota in Rats

#### Pharmacokinetics of Paeoniflorin in the Pseudo-Germ-Free (PGF) Rats

Fourteen SD rats were orally administered cefadroxil (100 mg/kg/day), oxytetracycline (300 mg/kg/day), and erythromycin (300 mg/kg/day) for 3 consecutive days to achieve PGF status. The remaining fourteen SD rats were orally administered saline equivalently. Two days after administration, pharmacokinetic experiments were performed. Rat colonies were collected on day 3 post-treatment and colony culture was performed on a nutrient agar culture medium to confirm the PGF status.

Prior to a single oral administration of paeoniflorin (20 mg/kg), rats were fasted overnight, but had free access to water. Venous blood samples (100 μL) were collected into 1.5 mL heparinized EP tubes at 0.00, 0.08, 0.17, 0.33, 0.5, 0.75, 1, 2, 4, 6, 10, and 12 h after administration of paeoniflorin. Plasma samples were immediately stored at 4°C until analysis.

#### Excretion of Paeoniflorin in Rats

Rats were kept in metabolic cages to collect urine and feces after oral administration paeoniflorin at the dose of 20 mg/kg. Then, urine samples and feces samples were collected at 0, 6, 12, 24, 48, 72, and 96 h.

### Carboxylesterase-Mediated Transformation of Paeoniflorin

#### Metabolic Transformation of Paeoniflorin in 18 Strains of Bacteria *in vitro*

After the activation and cultivation of 18 strains of bacteria (*B. longum, L. acidophilus, C. butyricum, B. breve, L. casei, S. aureus, S. epidermidis, E. aerogenes, E. coli, P. vulgaris, E. cloacae, E. cecorum, P. mirabilis, K. pneumonia, Sh. boydii, E. faecium, P. aeruginosa*, and *E. faecalis*), 10 μL of paeoniflorin solution was added to a 1 mL culture system with a final concentration of 100 μg/mL. Cultures were incubated at 37°C, followed by sample preparation as previously. After 24 h, the cultivation was terminated followed by determination of the concentrations of benzoic acid.

The DNA genome of *B. longum* (ATCC 15697), *L. acidophilus* (ATCC 4796), *S. aureus* (ATCC 6538), *B. breve* (ATCC 15700) were purified using the TIANamp Bacteria DNA Kit considering the manufacturers’ recommendations. Ten pairs of PCR primers were designed using primer premier5.0 and were provided in Table 1. The genes encoding carboxylesterases of these bacteria were amplified by PCR according to their genome template with primers, respectively. The agarose gel electrophoresis and gene sequencing method were performed to verify the sequences of these genes. Enzymes of PCR were used in the light of the manufacturers’ recommendations. All fragments were validated by Sanger 3730 sequencing.

**Table 1:** Sequences of primers in this study.

Primer name	Sequence (5′–3′)
SA1-F	ATGAGAATTAAAACACCGAGTCC
SA1-R	TGTTAAAGCATTGAAAAAGCGA
SA2-F	ATGCAGATAAAATTACCAAAACC
SA2-R	TTCTGACCAGTCTAATGACTCTAAA
SA3-F	ATGAAGATTAATACTACAGGTGGTC
SA3-R	TTGATATGTGCGATAATAGCGAC
SA4-F	TTTCCCAAGTATTTGTTGACCAG
SA4-R	ATGGAACATATTTTTAGAGAAGGAC
BB1-F	ATGAGTAATCTTGCCTCGTTCTG
BB2-R	TCAGGCTATCGAACAGTCCCAC
BB2-F	ATGGCACAGCCGCAGCCGTATTAC
BB2-R	TCATCTCAACGGAGCGAACGCCATA
BB3-F	ATGGCAATAGAACTGGCCAATCAT
BB3-R	TCAGTGTTGTTGCACGCGCTTG
BB4-F	GTGAGCGGGGCGGGGCGCATAC
BB4-R	TTATATTTCGCCGGAAACATGAG
BL1-F	CAGCACGCCAGAAGTAATGTCG
BL1-R	ATGAGCAAAGAAGCGAATGCCAG
LLA1-F	ATGCTTCCTATGGTTGTAATTAAAC
LLA1-R	TTTCTGTCTTTTAATCGTTTGATAT

#### Paeoniflorin Molecular Virtual Docking Analysis

Docking analysis between paeoniflorin and carboxylesterase were conducted using Discovery Studio Client software (v16.1.0.15350). The crystal structure of carboxylesterase 1R1D was obtained from the Protein Data Bank (PDB) database and the docking between paeoniflorin and carboxylesterase was performed using the CDOCKER approach as the docking algorithm for our docking study. During the docking process, the protein was kept rigid while the ligands were treated as fully flexible and a final minimization step was used to refine the docked positions. In addition, the parameters were set to the default values.

#### Effect of BNPP Inhibition on the Transformation of Paeoniflorin

Consistent with the above steps, the carboxylesterase inhibitor BNPP (0.1, 1, 3, and 10 mM) was added to the culture system at different concentrations with a final concentration of 1 mg/mL paeoniflorin. Then, the cultures were incubated at 37°C for 0, 2, 6, 12, and 24 h. The intestinal culture system without BNPP was used as a control group.

### Analysis of Microbial Diversity

Rat feces of the chronic unpredictable depression model group were collected after 2 weeks of treatment. Three groups, including the normal control group, the model control group, the paeoniflorin treatment group (oral, 20 mg/kg) were selected. Barcoded pyro sequencing analysis was performed on the V3 and V4 regions by 16S rRNA gene sequencing.

### Statistical Analysis

Statistical analyses were conducted using two-way ANOVA and Student’s *t*-test with GraphPad Prism Version 5 (GraphPad Software, La Jolla, CA, United States). The data are expressed as the means ± standard deviation. *P*-values less than 0.05 were considered statistically significant. DAS 3.0 was used to calculate the plasma pharmacokinetic parameters.

## Results

### Paeoniflorin Is One of the Major Components in Xiaoyao Wan

Ultra-performance liquid chromatography method was used to determine the content of paeoniflorin in Xiaoyao Wan. Supplementary Figure S2 shows the chromatogram of paeoniflorin in Xiaoyao Wan with the content of 4.8 mg/mL, which indicated that paeoniflorin would be one of the major components in Xiaoyao Wan.

### Paeoniflorin Has Antidepressant Efficacy in Rats

**FIGURE 1:**
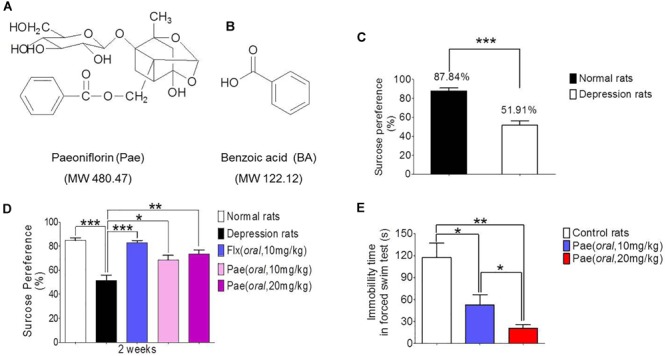
The antidepressant activity of paeoniflorin. **(A)** The chemical structure of paeoniflorin. **(B)** The chemical structure of benzoic Acid. **(C)** Sucrose preference tests after 8 weeks of stimulation in normal rats and depressed rats (*n* = 8). **(D)** Sucrose preference tests after 2 weeks of paeoniflorin treatment (*n* = 8). **(E)** Immobility time of rats in the forced swimming test (*n* = 5). ^∗^*P* < 0.05, ^∗∗^*P* < 0.01, ^∗∗∗^*P* < 0.001.

The chronic unpredictable stress model was established by continuous stimulation of SD rats for 8 weeks. The degree of depression was determined by the sucrose preference test. Figure 1C shows the results of the sucrose preference measurement. The sucrose preference value of normal rats (the control group) was 87.84%, however, the depressed rats (the model group) had a value of only 51.91%, which was significantly different from the control value (^∗∗∗^*P* < 0.001). Hence the chronic unpredictable stress depression model was successfully established.

The sucrose preference test results after 2 weeks of drug treatment are shown in Figure 1D. The sucrose preference value of the model group was lower than that of the normal group (^∗∗∗^*P* < 0.001), and the sucrose preference value of the fluoxetine group was significantly higher than that of the model control group (^∗∗∗^*P* < 0.001), which proves the reliability of this model. In addition, the sucrose preference value in the paeoniflorin-treated group (10 mg/kg and 20 mg/kg) was higher than that in the model group (^∗^*P* < 0.05 and ^∗∗^*P* < 0.01) and exhibited a dose-dependent response, suggesting paeoniflorin an antidepressant effect.

Moreover, SD rats were subjected to a forced swimming test to test the antidepressant activity of paeoniflorin. After 1 h of paeoniflorin treatment, SD rats were forced to swim for 6 min, and the immobility time of rats was recorded during the last 4 min as shown in Figure 1E. The immobility time of the paeoniflorin treatment group (10 and 20 mg/kg) was significantly higher than that of the control group (^∗^*P* < 0.05 and ^∗∗^*P* < 0.01) in a dose-dependent response manner to paeoniflorin (^∗^*P* < 0.05), indicating that paeoniflorin possessed antidepressant activities.

### Low Bioavailability of Paeoniflorin in Rats

**FIGURE 2:**
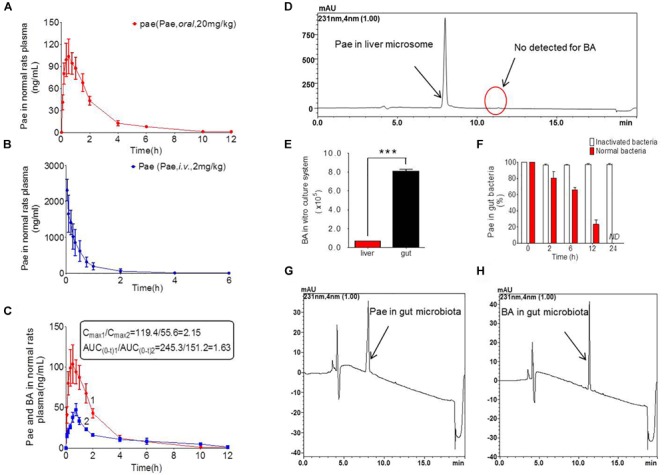
Pharmacokinetics study of paeoniflorin *in vivo* and *in vitro*. **(A)** Paeoniflorin concentration in the plasma of the normal rats after oral administration of paeoniflorin (20 mg/kg, *n* = 6). **(B)** Paeoniflorin concentration in the plasma of normal rats after intravenous administration of paeoniflorin (2 mg/kg, *n* = 5). **(C)** Paeoniflorin and benzoic acid in the plasma of the normal rats after oral administration of paeoniflorin (20 mg/kg, *n* = 6). **(D)** Determination of paeoniflorin and benzoic acid in the liver microsome after incubation for 2 h (*n* = 5). **(E)** Benzoic acid generation after incubation in gut microbial and liver tissue homogenate for 1 h (*n* = 5). **(F)** The content of paeoniflorin in the normal gut microbiota and the inactivated gut microbiota after incubation for 0, 2, 6, 12, and 24 h (*n* = 5). **(G,H)** Determination of paeoniflorin and benzoic acid in the gut microbiota *in vitro* before incubation and after incubation for 24 h (*n* = 5). ND, not detected. ^∗∗∗^*P* < 0.001.

The results of method validation were shown in the Supplementary Figure S1 and Supplementary Table S1. And all of the assay validation results were within the acceptable limits. Normal rats were orally administered of paeoniflorin (20 mg/kg) and intravenously administered of paeoniflorin (2 mg/kg). Their pharmacokinetic curves were plotted as shown in Figure 2A,B. The AUC_0-t_ of paeoniflorin was 245.29 ± 72.811 μg/L^∗^h; and 1062.211 ± 325.066 μg/L^∗^h (Table 2) after oral and intravenous administration, respectively. The absolute bioavailability of paeoniflorin was only 2.32%, which was calculated according to the following formula: F = AUCT⋅Div/AUCiv⋅DT × 100%. In the formula, AUC means drug concentration-time curve and D represents the dose to be administrated. In addition, iv and T represent the reference preparations of the intravenous injection and the test preparation, respectively.

**Table 2:** Pharmacokinetic parameters of paeoniflorin by intravenous and oral administration.

Parameters	Units	Intravenous	Oral
		2 mg kg^-1^ (*n* = 5)	20 mg kg^-1^ (*n* = 6)
Tmax	h	–	0.458 ± 0.195
*t*1/2z	h	2.056 ± 0.771	1.296 ± 0.474
Cmax	μg/L	–	119.355 ± 54.3
AUC (0-t)	μg/L^∗^h	1062.211 ± 325.966	245.292 ± 72.811
AUC (0-∞)	μg/L^∗^h	1067.296 ± 324.984	246.07 ± 72.811
MRT (0-t)	h	0.903 ± 0.122	1.996 ± 0.353
MRT (0-∞)	h	0.981 ± 0.07	2.035 ± 0.368
Vz	L/kg	6.091 ± 3.107	161.261 ± 82.928
CLz	L/h/kg	1.995 ± 0.512	88.379 ± 29.015

*Each value represents mean ± SD.*

Additionally, we detected benzoic acid in the blood after oral administration of paeoniflorin. The *t*_max_ of benzoic acid (0.792 h) was slightly delayed compared to that of paeoniflorin (0.458 h) which verified that benzoic acid might undergo a transformation from paeoniflorin in the gut before entering the blood (Figure 2C). The C_max_ of benzoic acid was 55.6 ng/mL with an AUC_0-t_ value of 151.2 μg/L^∗^h. Then the C_max_ and AUC_0-t_ of paeoniflorin were 2.15 and 1.63 times the values of benzoic acid, respectively. However, both values were quickly eliminated after the drugs were absorbed into blood. According to the chemical structure, paeoniflorin (Figure 1A) would be hydrolyzed and produces benzoic acid (Figure 1B) through ester bond fragmented. Furthermore, benzoic acid may be absorbed and enter the blood circulation.

### Gut Bacteria Hydrolyzed Paeoniflorin to Produce Benzoic Acid *in vitro*

Paeoniflorin was added to the incubation system of intestinal bacteria for cultivation, and the results are shown in Figure 2G,H. After a 24-h incubation, paeoniflorin was completely transformed to generate a new metabolite (Figure 2F) in the intestinal bacteria group compared with that in the inactivated control group, in which paeoniflorin was not metabolized, and no benzoic acid produced. The metabolite of paeoniflorin was identified to be benzoic acid based on the reference standard as well as the mass spectrometry information.

In addition, there was no benzoic acid produced when paeoniflorin was cultivated in the liver microsome system (Figure 2D). However, after paeoniflorin was added to the liver tissue homogenate for 1 h, paeoniflorin was metabolized into benzoic acid, but the production of benzoic acid in this system was significantly lower than that in the intestinal bacteria incubation system (^∗∗∗^*P* < 0.001) (Figure 2E). This result demonstrated that the metabolic enzyme, which making an influence on the transformation process, might be hardly expressed by the liver, meanwhile, exhibited lower activity than that in the gut. Thus, benzoic acid may be a characteristic metabolite of paeoniflorin produced by the gut microbiota.

### Pharmacokinetics of Paeoniflorin Mediated by the Gut Microbiota

**FIGURE 3:**
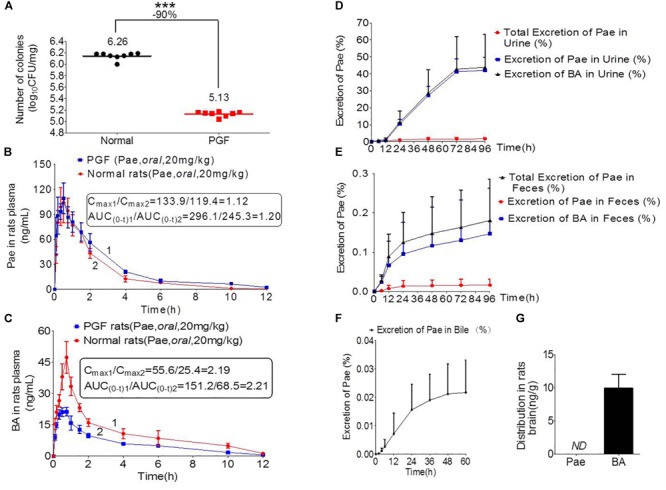
Pharmacokinetics study of paeoniflorin mediated by intestinal microbiota. **(A)** Number of colonies of normal and PGF rats. **(B)** Paeoniflorin concentration in the plasma of normal and PGF rats after oral administration of paeoniflorin (20 mg/kg, *n* = 6). **(C)** Benzoic acid in the plasma of the normal and PGF rats after oral administration of paeoniflorin (20 mg/kg, *n* = 6). **(D)** Excretion of paeoniflorin and benzoic acid after treatment with paeoniflorin (20 mg/kg) in normal rat urine (*n* = 6). **(E)** Excretion of paeoniflorin and benzoic acid after treatment with paeoniflorin (20 mg/kg) in normal rat feces (*n* = 6). **(F)** Excretion of paeoniflorin after treatment with paeoniflorin (20 mg/kg) in normal rat bile (*n* = 6). **(G)** Brain distribution of paeoniflorin and benzoic acid in the rats after oral treatment with paeoniflorin (20 mg/kg) at 1 h (*n* = 6). ND, not detected; PGF, pseudo-germ-free. ^∗∗∗^*P* < 0.001.

Figure 3A shows the number of bacterial colony forming units (CFUs) in the intestine of rats in the normal and PGF groups. The CFU in the PGF group was significantly lower than that in the normal group (^∗∗∗^*P* < 0.001), with an inhibition rate reaching 90%. These indicated that the PGF status was successfully established.

The normal and PGF rats were orally administered of paeoniflorin (20 mg/kg). Figure 3B,C shows the drug concentration-time curves of paeoniflorin and benzoic acid; at the same time, the pharmacokinetic parameters are shown in Table 3. The C_max_ and AUC_0-t_ of paeoniflorin in the plasma of the PGF group rats were 1.12 and 1.21 times higher than those in the plasma of the normal group, respectively (Figure 3B). Plasma benzoic acid was significantly lower in the PGF group than in the normal group (Figure 3C). Additionally, the values of C_max_ and AUC_0-t_ of plasma benzoic acid in the normal group were 2.19 and 2.21 fold higher than those in the PGF group (Figure 3C), respectively.

**Table 3:** Pharmacokinetic parameters of paeoniflorin and benzoic acid by oral administration in normal rats and rats pretreated with antibiotics.

Parameters	Units	Normal group		PGF group	
		Paeoniflorin	Benzoic acid	Paeoniflorin	Benzoic acid
Tmax	h	0.458 ± 0.195	0.792 ± 0.188	0.388 ± 0.136	0.597 ± 0.239
t1/2z	h	1.296 ± 0.474	2.555 ± 0.823	2.582 ± 1.614	2.006 ± 0.789
Cmax	μg/L	119.36 ± 54.3	55.58 ± 12.09	133.91 ± 48.55	27.62 ± 2.63
AUC (0-t)	μg/L^∗^h	245.29 ± 72.811	151.24 ± 29.59	296.08 ± 100.14	94.85 ± 19.82
AUC (0-∞)	μg/L^∗^h	246.07 ± 73.073	161.60 ± 40.10	312.15 ± 123.07	97.18 ± 20.23

*Each value represents mean ± SD.*

In addition, Figure 3D,E show the excretion rate of paeoniflorin and benzoic acid in urine and feces after oral administration of paeoniflorin (oral, 20 mg/kg). The 96-h cumulative excretion of paeoniflorin and benzoic acid was 1.66 and 42.13% in urine, as well as 0.016 and 0.15% in feces, respectively. The excretion of benzoic acid was significantly higher than that of paeoniflorin in urine and feces. The total excretion of paeoniflorin in urine and feces is shown in Table 4. To the contrary, the cumulative excretion rate of paeoniflorin in bile for 60 h was only 0.02% (Figure 3F). No benzoic acid was detected in rat bile, which suggested benzoic acid a metabolite from the gut bacteria.

**Table 4:** Cumulative fecal and urinary excretions of paeoniflorin after oral administration in rats at a dose of 20 mg kg^-1^.

Time (h)	Excretion rate of paeoniflorin (%)	
	Feces (%)	Urine (%)
0–6	0.0268 ± 0.0159	0.6821 ± 0.3209
6–12	0.0890 ± 0.0568	1.2565 ± 0.9639
12–24	0.1247 ± 0.0763	11.4988 ± 6.4969
24–48	0.1479 ± 0.0932	28.6890 ± 13.81
48–72	0.1630 ± 0.0948	42.8196 ± 19.2297
72–96	0.1805 ± 0.1055	43.7953 ± 19.5441

*Each value represents mean ± SD.*

Moreover, paeoniflorin was not detected in SD rat brains instead of a considerable number of benzoic acid at 2 h after oral administration of paeoniflorin (20 mg/kg) (Figure 3G). This result indicated that benzoic acid could cross the BBB and enter the brain, whereas paeoniflorin could not.

### Carboxylesterase-Mediated Transformation of Paeoniflorin in the Gut

**FIGURE 4:**
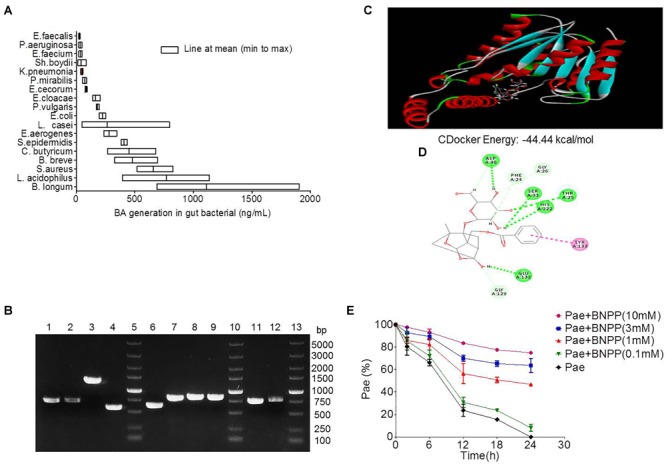
Transformation of paeoniflorin to benzoic acid mediated by carboxylesterase. **(A)** Production of benzoic acid in 18 strains of bacteria after the addition of paeoniflorin (*n* = 5). **(B)** The agarose gel electrophoresis results of *Staphylococcus aureus, Bifidobacterium breve, Lactobacillus acidophilus*, and *Bifidobacterium longum*. Lines 5, 10, 13: DNA marker; Lines 1–4: DNA fragments of *S. aureus*; Lines 6–9: DNA fragments of *B. breve*; Line 11: DNA fragment of *L. acidophilus*; Line 12: DNA fragment of *B. longum*. **(C)** Molecule docking principle between paeoniflorin and carboxylesterase 1R1D. **(D)** 2D schematic diagram of the binding of paeoniflorin to carboxylesterase. **(E)** Inhibition of the conversion from paeoniflorin to benzoic acid by incubation with BNPP *in vitro* (*n* = 5). BNPP, *bis-p*-nitrophenyl phosphate.

Eighteen strains of bacteria were incubated with paeoniforin for 24 h under anaerobic conditions, respectively. As shown in Figure 4A, all 18 strains of bacteria had the ability to convert paeoniflorin to benzoic acid, among these strains, *B. longum, L. acidophilus*, and *S. aureus* produced the most benzoic acid in place of *E. faecalis, B. breve*, and *S. aureus* mentioned in the previous study of albiflorin in our laboratory (Zhao et al., 2018). In addition, carboxylesterase gene fragments were discovered in the genomes of *B. longum, L. acidophilus*, and *S. aureus* using molecular biological analysis. We found the amino acid sequences functionalized as carboxylesterases through the NCBI database including *B. longum* (WP_007056568.1), *L. acidophilus* (EEJ76717.1), *S. aureus* (WP_001220807.1, WP_001165962.1, WP_000700912.1, WP_000400032.1), and *B. breve* (WP_003829800.1, WP_003829196.1, WP_003828396.1, WP_003828023.1). Here, we obtained gene sequences of the above proteins and the gene encoding carboxylesterases of these bacteria were amplified by PCR using primers. Then the agarose gel electrophoresis was applied to display the DNA fragments as well as identify the size of these genes shown up as Figure 4B. Additionally, gene sequencing of PCR products further validate that the gene sequences of these bacteria were in accordance with that searched in the NCBI database. This reveals that carboxylesterase gene might exist in these intestinal bacteria.

The crystal structure of carboxylesterase (PDB 1R1D) was obtained from the PDB database, followed by computer-aided docking analysis. Figure 4C shows the virtual docking principle between paeoniflorin and carboxylesterase. When paeoniflorin was in contact with carboxylesterase, the two molecules exhibited strong docking capabilities with a binding free energy of -44.44 kcal/mol. Figure 4D is a 2D schematic diagram of the binding of paeoniflorin to carboxylesteras. As shown in the figure, there were a large number of hydrophilic bonds (hydrogen bonds) in the active site of carboxylesterase, which might be the main binding force between paeoniflorin and carboxylesterase.

Additionally, the conversion of paeoniflorin to benzoic acid was inhibited when carboxylesterase inhibitors BNPP (0.1, 1, 3, and 10 mM) was added to the culture system, and the inhibition rates at 24 h were 8.2, 46.8, 63.7, and 74.9% (Figure 4E), respectively, which displayed a dose-dependence response.

In conclusion, the carboxylesterase of the gut microbiota might be an important bacterial enzyme that converts paeoniflorin into benzoic acid.

### Paeoniflorin Regulated the Composition of the Intestinal Microbiota

**FIGURE 5:**
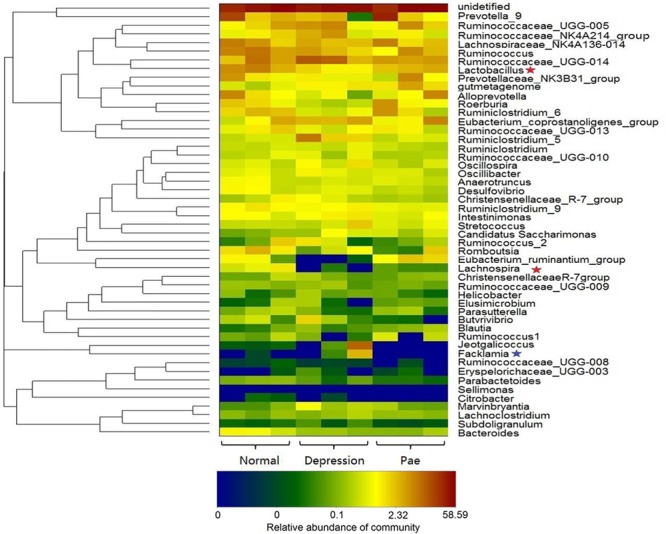
Bacterial composition analysis by 16S rRNA. The heat-map shows the top 50 bacterial genera with the most substantial change in abundance after the establishment of the depression model and paeoniflorin treatment. The color of the spot corresponds to the normalized and log-transformed relative abundance of genera. The change in color from blue to red represents corresponding colony abundance. ★The red pentagram represents bacteria with abundance increased after treatment with paeoniflorin. ★The blue pentagram represents bacteria with decreased abundance after treatment with paeoniflorin.

After 2 weeks of treatment with paeoniflorin in rats, feces samples were collected for analysis of intestinal flora composition. The pyrosequencing of the V3 and V4 regions was analyzed by 16S rRNA gene sequencing. Figure 5 is a heat map of the top 50 genera in the microbial composition after treatment with paeoniflorin. Among the 50 genus strains, the abundance of five species (*Intestinimonas, Oscillibacter, Ruminiclostridium_5, Eubacterium_coprostanoligenes_group*, and *gutmetagenome*) was increased in the depression group compared with that in the normal group. By contrast, the abundance of six strains (*Bacteroides, Eubacterium_ruminantium_group, Lachnospira, Ruminococcaceae_UCG-013, Roseburia*, and *Lactobacillus*) was decreased. *Oscillibacter* (Naseribafrouei et al., 2014) is reportedly associated with depression.

Moreover, after the treatment group of paeoniflorin, the abundance of intestinal microflora in the depression model group was improved to a certain degree compared with that in the control group, including the abundance of four species (*Lactobacillus, Lachnospira, Eubacterium_ruminantium_group*, and *Roseburia*) was increased; however, the abundance of four species of bacteria (*Facklamia, Jeotgalicoccus, Ruminiclostridium_5*, and *Eubacterium_coprostanoligenes_group*) was decreased. *Lachnospira* has been used to prevent the occurrence of preschool asthma phenotypes (Stiemsma et al., 2016) and *Roseburia* has the potential to treat inflammatory bowel disease (Zhu et al., 2018). In contrast, *Facklamia* is a rarely reported cause of clinical infection (Rahmati et al., 2017). *Lactobacillu* inhibits the growth and toxicity of the fungal pathogen Candida albicans and reduces its pathogenicity (Vilela et al., 2015).

## Discussion

Xiaoyao Wan is a commonly used Chinese medicine for soothing the liver, strengthening the spleen, regulating qi and reducing stagnation. This medicine has been widely used to treat various forms of depression. Our study indicated that paeoniflorin, a main component of Xiaoyao Wan, showed significant antidepressant activity in a chronic unpredictable depression model and forced swimming tests. Consistent with the previous work (Takeda et al., 1995), the oral bioavailability of paeoniflorin was low at only 2.3% in this study; therefore, we inferred that the esterase-catalyzed hydrolysis of paeoniflorin could also generate by the gut bacteria as similar as that of the report of albiflorin (the isomer of paeoniflorin in Xiaoyao Wan) (Zhao et al., 2018). Interestingly, we found benzoic acid in rat plasma after orally administrated of paeoniflorin. Moreover, in the paeoniflorin excretion experiments, paeoniflorin was mainly excreted in the form of benzoic acid in urine and feces. By contrast, the excretion of paeoniflorin in prototype drug was minimal, suggesting that paeoniflorin was mostly metabolized, and benzoic acid might be the main metabolite of paeoniflorin. However, its cumulative excretion was only approximately 50%, suggesting the presence of other metabolites that were not detected in this study likely due to their lower levels. Moreover, the excretion of benzoic acid in urine was significantly higher than that in feces, indicating good absorption of benzoic acid; in another word, benzoic acid was absorbed after the metabolism of paeoniflorin, and excreted via urine. In addition, limited quantity of paeoniflorin was excreted in the form of a prototype drug through bile, demonstrating that very little paeoniflorin could be metabolized via the liver.

Furthermore, *in vitro* incubation experiments revealed that benzoic acid might be a characteristic metabolite of paeoniflorin in the gut microbiota. According to pharmacokinetic studies of paeoniflorin mediated by the gut microbiota, the benzoic acid level in the blood of the PGF group was approximately 48% lower than that in the blood of the normal group, while paeoniflorin in the blood of the PGF group was increased by approximately 21%. This finding shows that antibiotics led to a decrease in the abundance of the gut microbiota, which weakened its hydrolysis action on paeoniflorin. This intervention can promote the absorption of paeoniflorin and reduce the metabolic conversion of paeoniflorin to benzoic acid, which might provide an explanation for the low bioavailability of paeoniflorin after oral administration.

Based on the present research, paeoniflorin does not penetrate the BBB; however, benzoic acid can pass through the BBB and enter the brain. Benzoic acid is a DAAO inhibitor that inhibits D-amino acid degradation. DAAO inhibitors can slow down the metabolism and increase the synaptic concentration of D-amino acid of the NMDAR co-agonist, indirectly increasing NMDAR activity. Benzoate improves cognitive function in patients with chronic schizophrenia and early Alzheimer’s disease, supporting the efficacy of DAAO inhibitors in promoting cognitive ability (Lane et al., 2013; Terry-Lorenzo et al., 2014; Lin et al., 2017). These results further proved that benzoic acid may enter the central nervous system to exert antidepressant effects.

Next, we investigated what factors catalyzed this transformation (from paeoniflorin to benzoic acid). With culture experiments of 18 strains of intestinal bacteria *in vitro*, molecular docking experiments, specific inhibitor experiments and molecular biological analysis, we found that carboxylesterase is one of the key enzymes that mediated this transformation process. Interestingly, carboxylesterase made an essential action in the transformation process, but the interaction between paeoniflorin and the gut bacteria is a little different from the isomer of paeoniflorin (albiforin) in our previous work (Zhao et al., 2018); besides, the subtype of carboxylesterase acting on the transformation of paeoniflorin or albiforin was diverse which was certified by the molecular docking analysis (Carboxylesterase 1R1D for paeoniflorin; Carboxylesterase 1AUO for albiflorin). Our investigations exemplify that isomer compounds like paeoniflorin and albiforin exhibit similar structure, whereas possess various activities on pharmacodynamic mechanism.

In addition, microbial diversity analysis demonstrated that paeoniflorin can alleviate depressive symptoms through the regulation of gut microbiota abundance, increasing the abundance of probiotics. Moreover, *L. acidophilus* belongs to *Lactobacillus* and possesses strong ability to convert paeoniflorin to benzoic acid. Additionally, the abundance of *Lactobacillus* was regulated after treatment with paeoniflorin in depressed rats. This finding indicates that the interaction between paeoniflorin and the gut microbiota further promoted antidepressant action of paeoniflorin.

## Conclusion

In summary, the present study found that the degradation of the gut microbiota may be one of the main reasons for the low bioavailability of paeoniflorin. Benzoic acid, the gut characteristic metabolite of paeoniflorin, can be absorbed into blood and penetrate the BBB and enter the central nervous system relieving depressive behaviors. Moreover, the interaction between paeoniflorin and the gut microbiota can further enhance its antidepressant effects. In conclusion, this paper provides new insight and approaches in regard to natural products with good efficacy, poor absorption, and unclear mechanisms of action, especially for drugs acting on the central nervous system, and has a certain guiding significance.

## Data Availability

All datasets generated for this study are included in the manuscript and/or the Supplementary Files.

## Author Contributions

L-XS, YW, and J-DJ conceptualized experiments and analysis. Z-XZ and J-BY designed the experiments. J-BY and L-BP performed the animal experiments. J-BY and JF performed the biotransformation study. RP and J-BY performed the molecular biology study. S-RM, Z-WZ, and LC performed the plasma pharmacokinetics method validation. PH modified the language. J-BY and YW analyzed the data and wrote the manuscript. All authors read and approved the final version of the manuscript.

## Conflict of Interest Statement

The authors declare that the research was conducted in the absence of any commercial or financial relationships that could be construed as a potential conflict of interest.

## Abbreviations

BBB: blood–brain barrier
BNPP: *bis-p*-nitrophenyl phosphate
DAAO: D-amino acid oxidase
NMDAR: *N*-methyl-D-aspartate receptor
PGF: pseudo-germ-free
