# Supplementary material for: Gut Microbiota-Based Pharmacokinetics and the Antidepressant Mechanism of Paeoniflorin
Source: Front Pharmacol. 2019 Mar 20;10:268. doi: 10.3389/fphar.2019.00268 (PMC6435784; doi:10.3389/fphar.2019.00268)
Supplement: Supplementary file 1 [file Table_1.DOCX]

**Supplementary Material**

**Validation of Paeoniflorin and Benzoic acid in plasma by LC-MS/MS**

**Calibration standards and QC preparation**

Plasma (90 µL) was mixed with 10 µL of working solutions of the calibration standards and 10 µL of IS (5 ng/mL), then precipitated with 300 µL of acetonitrile. After vortexing, samples were centrifuged at 14,800 rpm for 10 min. The standard curve concentrations of paeoniflorin were as follows: 2.5, 5, 12.5, 50, 250, 1250, 2500, and 5000 ng/mL. The standard curve concentrations of benzoic acid were 1, 2, 5, 20, 100, 500, 1000, and 2000 ng/mL, respectively. And QCs were prepared similarly. The concentrations of LLOQ and QC for paeoniflorin were 2.5 ng/mL (lower limit of quantitation, LLOQ), 5 ng/mL (low concentration of quality control, LQC), 250 ng/mL (median concentration of quality control, MQC), and 4000 ng/mL (high concentration of quality control, HQC). The concentrations of LLOQ and QC for benzoic acid were 1 ng/mL (lower limit of quantitation, LLOQ), 2 ng/mL (low concentration of quality control, LQC), 100 ng/mL (median concentration of quality control, MQC), and 1600 ng/mL (high concentration of quality control, HQC).

**Method validation**

The method was validated in terms of specificity, linearity and sensitivity, intra- and inter-day accuracy and precisions, recovery, matrix effect and stability according to the currently accepted Chinese State Food and Drug Administration (SFDA) bioanalytical method validation guidelines.

**Specificity**

The specificity of the method was assessed by analysing six batches of blank rat plasma, blank plasma samples spiked with paeoniflorin, benzoic acid and IS, and plasma samples obtained from the rats after oral administration, respectively.

**Linearity and sensitivity**

Linearity was determined by plotting the peak area ratio of analytes to internal standard against the analytes concentrations of the calibration standards with weighted (1/*c*) least square linear regression. And the calibration curve including eight concentration levels of samples covering the designated range and lower limit of quantification (LLOQ).

**Precision and Accuracy**

The inter- and intra-day precision and accuracy were carried out by analyzing repeated quality control (QC) samples (n=5) at LLOQ and low, middle, high QC concentration levels on three consecutive validation days. The precision and accuracy was expressed as the RSD(%) and RE(%), respectively.

**Recovery and Matrix Effect**

Recovery of analytes was calculated by comparing the analytes peak area ratios from five replicates QC samples at LLOQ and low, medium, and high concentrations that in the post-treatment spiked samples to that acquired from pre-treatment spiked samples. The matrix effects were investigated by comparing the peak areas of the analytes dissolved in post-extracted blank plasma at QC levels with that of the pure standard solutions containing same concentration of analytes.

**Stability**

The stability of analytes in rat plasma was investigated by analyzing QC samples stored under different conditions including plasma at ambient temperature for 24 h, −20◦C for 72 h, and after three freeze-and-thaw cycles, as well as plasma samples prepared in the autosampler for 24 h at 4◦C

**Results**

Typical mass spectra of paeoniflorin and benzoic acid in rat plasma were shown in the Figure S1. Paeoniflorin, benzoic acid and IS were detected at 3.806 min, 4.542 min and 4.464 min, respectively. No significant interference with the analytes was observed with the endogenous material in the rat plasma.

The calibration curves of the analytes were linear with correlation coefficients (r) > 0.99, and the linear ranges were 2.5-5000 ng/mL for paeoniflorin and 1-2000 ng/mL for benzoic acid, respectively. The LLOQs for paeoniflorin and benzoic acid were detected to be 2.5 and 1.0 ng/mL, respectively.

The extracted recovery and matrix effect were in the range of 99.85–104.16% and 96.88–102.54%, respectively, with RSD meeting the bioanalytical requirements. And all of the assay validation parameters of the stability results were within the acceptable limits.

The RSD(%) and RE(%) values were typically <20% and <15% for all analytes. These data demonstrates that the developed method was reliable and reproducible for the quantitative analysis.

| TABLE S1 \| Validation results of Paeoniflorin and Benzoic acid in plasma by LC-MS/MS (*n* = 5) | | | | | | | | | |
| --- | --- | --- | --- | --- | --- | --- | --- | --- | --- |
|  | **Paeoniflorin** | | | |  | **Benzoic acid** | | | |
|  | **LLOQ** | **LQC** | **MQC** | **HQC** |  | **LLOQ** | **LQC** | **MQC** | **HQC** |
| **PRECISION AND ACCURACY** | | | | | | | | | |
| **Inter-day** | | | | | | | | | |
| **Batch 1** | | | | | | | | | |
| Mean(ng/mL) | 2.39 | 4.83 | 257.11 | 3865.18 |  | 0.93 | 2.07 | 95.07 | 1611.13 |
| RE(%) | -4.43 | -3.40 | 2.84 | -3.37 |  | -2.63 | 1.46 | -1.97 | 0.28 |
| RSD(%) | 9.90 | 9.16 | 4.11 | 1.97 |  | 14.63 | 5.29 | 9.53 | 1.34 |
| **Batch 2** | | | | | | | | | |
| Mean(ng/mL) | 2.35 | 5.17 | 267.38 | 3945.57 |  | 1.05 | 2.09 | 94.43 | 1474.58 |
| RE(%) | -5.82 | 3.34 | 6.95 | -1.36 |  | 1.83 | 1.80 | -2.23 | -3.14 |
| RSD(%) | 7.99 | 3.24 | 7.02 | 3.56 |  | 8.42 | 6.68 | 3.19 | 4.42 |
| **Batch 3** | | | | | | | | | |
| Mean(ng/mL) | 2.53 | 5.21 | 267.22 | 3897.99 |  | 0.92 | 2.04 | 98.60 | 1700.87 |
| RE(%) | 1.39 | 4.22 | 6.89 | -2.55 |  | -3.16 | 0.76 | -0.56 | 2.52 |
| RSD(%) | 2.44 | 6.23 | 1.62 | 2.38 |  | 14.35 | 6.23 | 8.67 | 3.30 |
| **Intra-day** | | | | | | | | | |
| Mean(ng/mL) | 2.43 | 5.07 | 263.9 | 3902.91 |  | 0.97 | 2.07 | 96.04 | 1595.53 |
| RE(%) | -2.95 | 1.39 | 5.56 | -2.43 |  | -3.30 | 3.36 | -3.96 | -0.28 |
| RSD(%) | 7.57 | 6.98 | 4.83 | 2.68 |  | 13.04 | 5.75 | 7.40 | 6.73 |
| **RECOVERY(%)** | | | | | | | | | |
| Mean | 99.85 | 102.50 | 101.88 | 104.16 |  | 100.41 | 99.85 | 102.54 | 96.88 |
| RSD(%) | 3.07 | 1.10 | 5.29 | 2.75 |  | 10.40 | 3.34 | 5.64 | 6.17 |
| **MATRIX EFFECT(%)** | | | | | | | | | |
| Mean | 98.37 | 102.53 | 101.44 | 103.59 |  | 100.15 | 104.49 | 106.05 | 98.59 |
| RSD(%) | 3.81 | 2.00 | 6.86 | 3.27 |  | 9.62 | 7.99 | 6.03 | 5.47 |
| **STABILITY OF PLASMA BEFORE PREPARATION** | | | | | | | | | |
| **At ambient temperature for 24 h** | | | | | | | | |  |
| Mean(ng/mL) | 2.55 | 5.10 | 244.85 | 3512.49 |  | 1.06 | 2.12 | 97.05 | 1604.02 |
| RE(%) | 2.01 | 2.08 | -2.06 | -12.19 |  | 6.36 | 5.93 | -2.95 | 0.25 |
| RSD(%) | 1.79 | 2.21 | 0.98 | 6.07 |  | 8.15 | 4.94 | 1.88 | 5.21 |
| **At -20℃ for 72 h** | | | | | | | | |  |
| Mean(ng/mL) | 2.70 | 5.21 | 255.45 | 4196.85 |  | 0.96 | 2.09 | 101.09 | 1693.15 |
| RE(%) | 8.08 | 4.12 | 2.18 | 4.92 |  | -3.90 | 4.54 | 1.09 | 5.82 |
| RSD(%) | 11.70 | 4.31 | 3.45 | 2.06 |  | 10.06 | 3.57 | 2.81 | 3.17 |
| **After three freeze-and-thaw cycles** | | | | | | | | | |
| Mean(ng/mL) | 2.46 | 5.10 | 262.65 | 4090.03 |  | 0.94 | 2.04 | 95.00 | 1631.40 |
| RE(%) | -1.46 | 1.97 | 5.06 | 2.25 |  | -5.96 | 2.24 | -5.00 | 1.96 |
| RSD(%) | 4.57 | 2.36 | 3.40 | 2.61 |  | 10.37 | 1.43 | 3.98 | 3.70 |
| **STABILITY OF PLASMA AFTER TREATMENT IN THE AUTOSAMPLER FOR 24h AT 4℃** | | | | | | | | | |
| Mean(ng/mL) | 2.61 | 5.13 | 260.30 | 4088.06 |  | 0.94 | 2.21 | 101.31 | 1651.41 |
| RE(%) | 4.30 | 2.58 | 4.12 | 2.20 |  | -5.88 | 10.49 | 1.31 | 3.21 |
| RSD(%) | 15.14 | 4.03 | 3.86 | 4.03 |  | 3.85 | 7.54 | 4.00 | 1.29 |

**Figure**

**Figure S1**


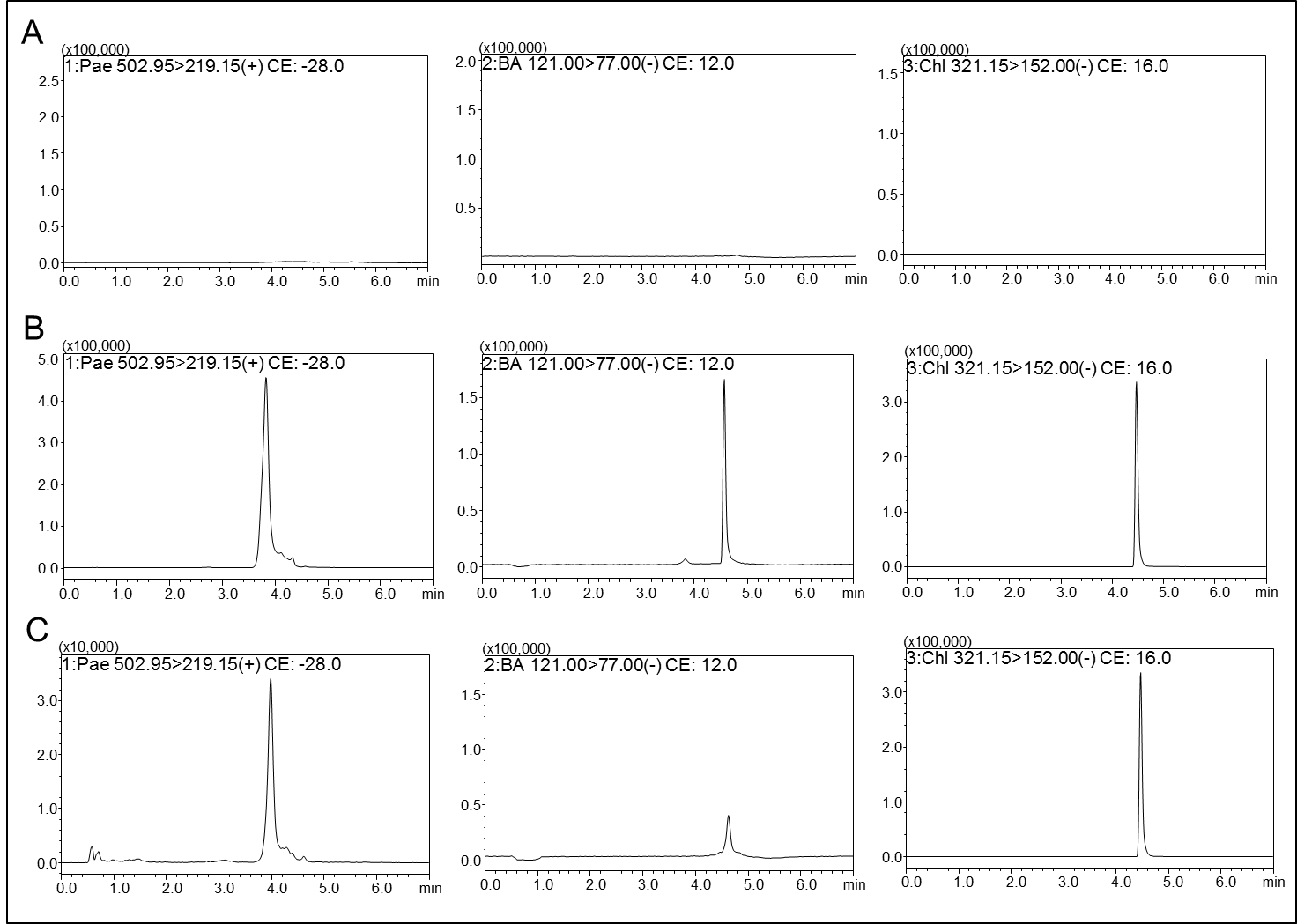


**Figure Legends:**

Figure S1: Typical mass spectra of paeoniflorin and benzoic acid in rat plasma (A: blank plasma; B: blank palsma spiked with paeoniflorin, benzoic acid and IS; C: plasma sample at 1 h after administration of paeoniflorin).

**Figure**

**Figure S2**


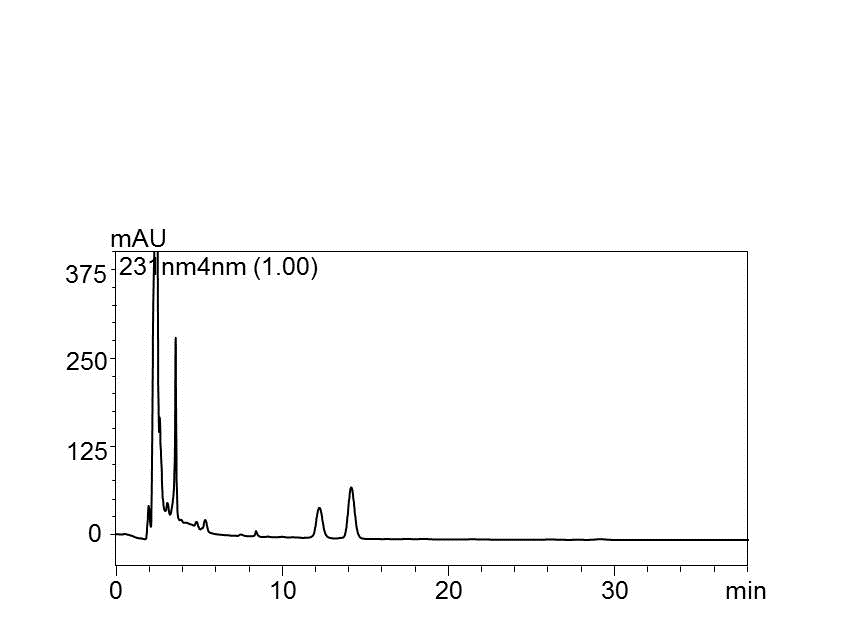


Paeoniflorin

(4.8 mg^.^g^-1^)

**Figure Legends:**

Figure S2: Determination of paeoniflorin in Xiaoyao Wan by UFLC.
